# Spatiotemporal patterns of non-genetically modified crops in the era of expansion of genetically modified food

**DOI:** 10.1038/srep14180

**Published:** 2015-09-18

**Authors:** Jing Sun, Wenbin Wu, Huajun Tang, Jianguo Liu

**Affiliations:** 1 Center for Systems Integration and Sustainability, Michigan State University, East Lansing, MI 48823, the United States; 2 Key Laboratory of Agri-informatics, Ministry of Agriculture/Institute of Agricultural Resources and Regional Planning, Chinese Academy of Agricultural Sciences, Beijing, 100081, P.R. China

## Abstract

Despite heated debates over the safety of genetically modified (GM) food, GM crops have been expanding rapidly. Much research has focused on the expansion of GM crops. However, the spatiotemporal dynamics of non-genetically modified (non-GM) crops are not clear, although they may have significant environmental and agronomic impacts and important policy implications. To understand the dynamics of non-GM crops and to inform the debates among relevant stakeholders, we conducted spatiotemporal analyses of China’s major non-GM soybean production region, the Heilongjiang Province. Even though the total soybean planting area decreased from 2005 to 2010, surprisingly, there were hotspots of increase. The results also showed hotspots of loss as well as a large decline in the number and continuity of soybean plots. Since China is the largest non-GM soybean producer in the world, the decline of its major production region may signal the continual decline of global non-GM soybeans.

Genetically modified (GM) crops continue to expand rapidly around the world, which has resulted in heated debates on a range of relevant issues such as health hazard and food safety^1^,^2^. Much research has been conducted on the expansion of GM crops, but little has been done on the spatial patterns of non-genetically modified (non-GM) crops. In the era of rapid expansion of GM food, analysing spatial patterns is essential to understand where non-GM crops are still grown, where they are increasing, and where they are declining. Information on hotspots of gain and loss of non-GM crops is crucial for policy makers, farmers, and other relevant stakeholders in their decision-making processes.

An excellent example is soybeans, which are an important crop for vegetable oil and protein, and one of the most important commodities in the global trade of food. Soybeans were originally domesticated in China approximately 3,100 years ago^3^, but China has recently become the largest soybean importer, where more than 80% of soybeans consumed were imported^4^,^5^, primarily from the United States and Brazil. Large-scale soybean cultivation began in the United States during the 1940s and 1950s^6^, and since 1996, GM soybeans have gradually dominated this market. By 2012, the total soybean production in the United States was measured at 82.0 million tons, worth approximately $24.6 billion; 93% of this production was GM^7^. Notably, 36.6 million tons (whole soybeans) of the total production were exported; China was the largest buyer at an estimated 25.9 million tons, or $14.9 billion^8^. In 2012, Brazil surpassed the United States as the largest soybean producer and exporter, producing 83.5 million tons and exporting 38.4 million tons^8^. China is the largest consumer of Brazil’s soybeans, purchasing about 23.7 million tons (whole soybeans) for approximately $12 billion in 2012^8^,^9^.

Apparently, as the top two soybean producers in the world, the United States and Brazil are closely associated with China, their largest customer. In response to the increased domestic demand for soybean oil and soybean meal induced by a large population and economic growth, China further increased its soybean import. Chinese soybean imports exceeded exports in 1995 and have been growing since. The imports reached 55 million tons in 2010, accounting for 56% of the world’s total soybean exports^8^,^9^ (Fig. 1). Considering possible environmental and social consequences, as well as food safety concerns, China has banned GM soybean cultivation, but still allows the import of GM soybeans. The proportion of GM soybeans also experienced a rapid increase after 1996^5^,^10^. The price of domestic non-GM soybeans cannot compete with that of imported GM soybeans, thereby leading to a decline in the amount of area devoted to soybean production in China^11^,^12^.

Throughout China, soybean lands are generally small and fragmented, and difficult to inventory either by fieldwork or remote sensing techniques. The exception is the traditional leading soybean production region – Heilongjiang Province in northeast China (Fig. 2). Covering a total area of 473,000 km^2^, Heilongjiang (121°11′–135°05′ E, 43°25′–53°33′ N) is a main agricultural production province, where soybeans, corn, and rice are three major crops. As the lead soybean-growing region, its production accounted for approximately one-quarter to one-third of the nation’s annual soybean production over the past decade^9^,^13^. Likewise, affected by the rising import of GM soybeans, the non-GM soybean planting area in Heilongjiang decreased from 43,722 km^2^ in 2005 to 35,384 km^2^ in 2010, with corn replacing soybeans as the dominant crop in Heilongjiang^14^,^15^. Conventional studies were based on the government’s statistical data. However, little has been done on the spatial and temporal analyses of soybean production in China, including Heilongjiang.

Information on the spatiotemporal dynamics of soybean production has important scientific value and far-reaching policy implications. For instance, because soybeans fix nitrogen and other crops cannot, the conversion from soybeans to other crops such as corn requires the application (and often overuse) of much more fertilizer. Thus, the conversion causes more severe environmental problems (e.g., eutrophication in freshwater ecosystems)^16^. The spatiotemporal information can be used to reflect changes of environmental impacts in space and time. Moreover, considering that the growing season required by corn is longer than soybeans, it leaves corn more vulnerable than soybeans in extreme weather, such as cold weather in late spring. The spatiotemporal information indicates the distribution of potential vulnerability of crops to extreme weather events. The same information also indicates the potential location and demand for new corn varieties that require a short growing season, which enhances the ability to cope with extreme weather events. This is of particular importance for regions of high latitude like Heilongjiang where temperature is a main determinant of crop cultivation.

Because more than 80% of China’s soybean consumption now depends on soybean imports, spatiotemporal dynamics of soybean production in China can lead to changes in land cover and land use in soybean-exporting countries such as Brazil^17^,^18^. A decrease of soybean production in China makes it become even more reliant on soybean imports. This may lead to more deforestation in the Amazon rainforest in the future. Although the implementation of forest conservation policies like the Soy Moratorium (an agreement that forbids major soybean traders to purchase soybeans grown on lands cleared after July 2006 in the Brazilian Amazon) has slowed the rate of forest clearing, the moratorium will expire soon and the future is uncertain^19^. Large areas of the Cerrado (tropical savannah) in Brazil have also been converted to soybean lands, which poses environmental problems, such as increased greenhouse gases emissions. Because soybean productivity (yield per hectare) varies throughout Heilongjiang (Supplementary Table 1), spatial dynamics of soybean production may affect the amount of production and imports. For example, soybean productivity in Mudanjiang and Hegang is the highest and lowest, respectively (Supplementary Table 1). A reduction of 10,000 ha of soybean lands in Mudanjiang would lead to a reduction of 22,000 tons of soybeans, while the same reduction of soybean lands in Hegang would cause only a reduction of 15,000 tons of soybeans. Thus, information on the spatial distribution of soybean lands can help predict total soybean production in Heilongjiang and, thus, help predict the demand for soybeans from soybean-importing countries (and ultimately land use and land cover changes in soybean-exporting countries).

To address the urgent need for understanding the spatiotemporal dynamics of non-GM soybeans in China, we used the major cropland data layers of 2005 and 2010 interpreted from satellite data to map the changes in soybean planting areas in Heilongjiang. We also measured the spatial patterns of the changes at multiple spatial scales.

## Results

### Hotspots of soybean changes in planting areas

The dynamics of soybean planting areas are complex (Fig. 3). Although the total soybean planting area has decreased, surprisingly there were hotspots of soybean gain, which mainly occurred in the prefectures of Qiqihar and Harbin (Fig. 3a). Hotspots of soybean loss were relatively extensive, and there were some highly concentrated areas in the prefectures of Jiamusi, Qitaihe, and Shuangyashan (Fig. 3b).

### Temporal changes in frequency profile of plot sizes

The profiles in Fig. 4 show the soybean area (amount of area planted in soybeans) in 2005, soybean area in 2010, soybean area loss (reduced amount of area planted in soybeans), and soybean area gain (increased amount of area planted in soybeans) in relation to their soybean area densities for three spatial scales. The soybean area density is the proportion of soybean planted area in a given region, and the soybean area refers to the sum of soybean pixels with the same density. Two vertical dashed lines, soybean area densities of 0.6 and 0.9, were superimposed to assist analysis. The density of 0.6 was to denote relatively continuous soybean planted areas, based on the percolation theory^20^. The density of 0.9 was selected to indicate a soybean-dominated area, which denotes highly continuous soybean plots^21^.

In Fig. 4a–c, we note that all the profiles for soybean area in 2005 and soybean area in 2010 are tailed to the right, but skewed to the left. The profile of soybeans in 2005 is higher than that of soybeans in 2010 in all the windows, which indicates a net loss of soybean planting area during the study period. In addition, the tail becomes shorter when the window size gets larger, and this tendency is obvious in Fig. 4b,c. The tails of soybean area in 2005 are longer than those of soybean area in 2010 in Fig. 4b,c, indicating that large continuous soybean croplands became rare from small to large scales. In Fig. 4d–f, all the profiles for soybean gain and loss have a shape similar to the distributions of soybean areas in 2005 and 2010. The tails of soybean loss are higher and longer than those of soybean gain, except for the cases when soybean area density is 0.025, which may show some small net increases in soybean planting area.

## Discussion

**Figure 2:**
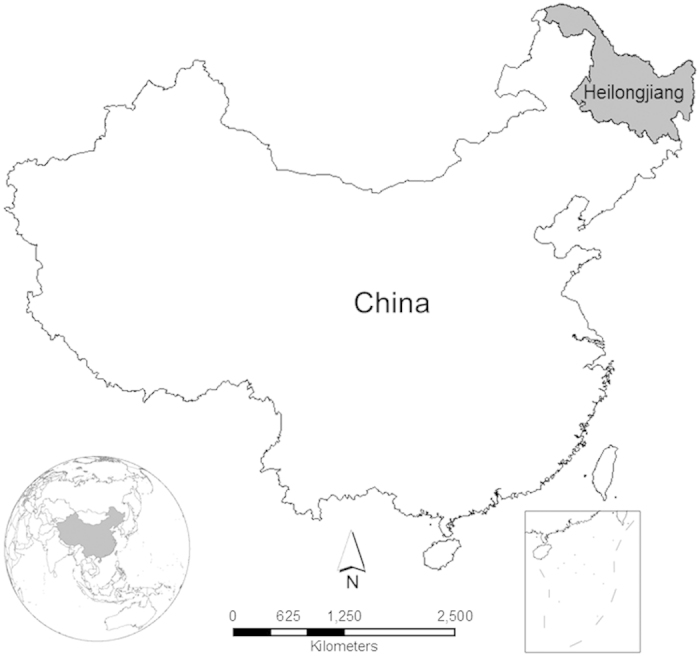
Map of China showing location of Heilongjiang Province. The map was generated by the software ArcGIS 10^42^.

**Figure 3:**
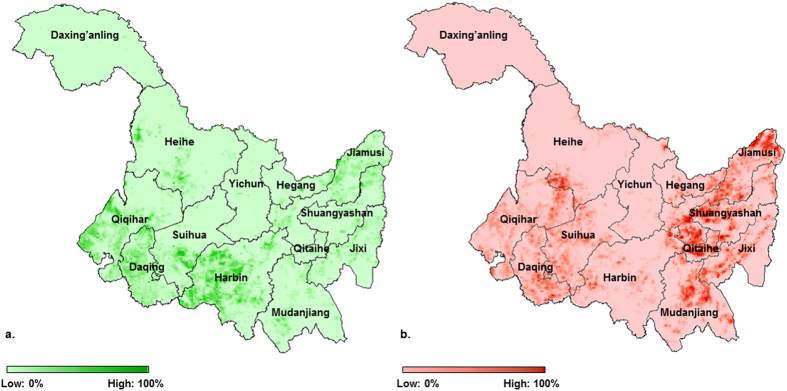
Hotspots of soybean dynamics from 2005 to 2010 in Heilongjiang Province, China. (**a**) hotspots of soybean gain. (**b**) hotspots of soybean loss. Names of prefecture-level regions are also shown. Colour bars describe the change rate of soybean dynamics from light colour (change rate: 0%) to dark colour (change rate: 100%). Because the pixel-level results of change detection (Supplementary Fig. 3) are not very informative and usually too scattered to visualize, they were normalized to change rate following a spatial smoothing technique^46^. Specifically, the spatial resolution of the original 250 m was reduced to 2500 m by superimposing 10 pixels × 10 pixels blocks (i.e., one block is composed of 100 pixels) on the entire region and then averaging the binary values within each block. The binary values in (**a**) equal 1 for the soybean gain pixels and 0 for other pixels and in (**b**) equal 1 for the soybean loss pixels and 0 for other pixels. A kernel smoother was applied to the normalized results and created change rate surfaces that portray the hotspots of soybean dynamics. The map was generated by the software ArcGIS 10^47^.

**Figure 4:**
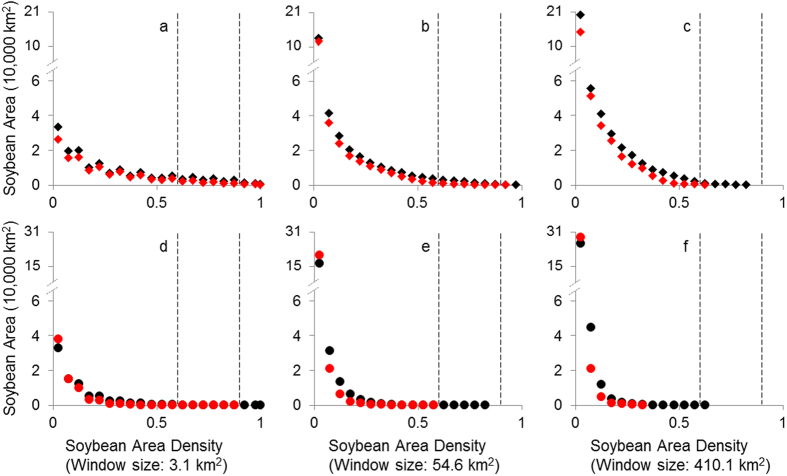
Soybean area 2005 (dark diamonds), soybean area 2010 (red diamonds), soybean area loss (dark circles), and soybean area gain (red circles) in relation to their soybean area density for three window sizes (3.1 km^2^, 45.6 km^2^, and 410.1 km^2^) in Heilongjiang Province, China. Two vertical dashed lines, 0.6 and 0.9, were superimposed to assist analysis. Note: to improve the visibility, we grouped the range of soybean area density (0, 1) into 20 equal-width intervals and used the midpoint to mark each interval, i.e. 0.025, 0.075, 0.125 ... 0.095, except for soybean area density = 1. Also, because soybean area density = 0 represents the non-soybean area, we did not plot it in the figure.

Unlike other land cover types such as forest, the dynamics of agricultural land are relatively fast and complex. In Heilongjiang, soybean loss was extensive, primarily due to the changes in land cover and land use. Specifically, the changes include the conversion from soybeans to corn, rice (Supplementary Fig. 1), or other rain-fed crops, because the profitability of other crops is higher than that of soybeans^15^,^22^. Some soybean lands have been converted to other land cover types, such as forest. However, there are several potential reasons for the accompanied soybean gain. For instance, the soybean gain in Harbin (Fig. 3a) may be due partially to the cold and snowy spring that occurred locally in 2010. This extreme weather event in Harbin led to the latest growing season in the past decade, while the growing season in other regions began at about the average time based on historical records (Supplementary Table 2). Because Heilongjiang is the most northeastern province (Fig. 2), the high latitude results in the temperature becoming a determinant in decisions about crop planting. Soybeans have a relatively shorter growing period compared with corn, and, therefore, they were chosen in Harbin as an alternative in case of a cold and snowy spring in years like 2010. During a survey across the entire Heilongjiang in 2013, we found that soybeans were still planted extensively in Qiqihar (Fig. 3a). This is because Qiqihar has one of the most favourable environments in Heilongjiang, which includes productive soils, and suitable precipitation and temperature for soybean growth^23^. We also found that, historically, the level of fertilizer use was relatively low in Qiqihar, and the use of pesticides and herbicides was limited. The high soybean yields and low costs (also including labour) may also have partially contributed to the soybean gain that occurred in Qiqihar. The soybean gain in other regions was heterogeneous but relatively low. The major cropland area (including corn, rice, and soybeans) in Heilongjiang increased from 113,220 km^2^ in 2005 to 142,500 km^2^ in 2010^13^, where most of the increased areas were converted from abandoned lands, as well as some wetland, grassland, or even forest^24^,^25^. It is common for abandoned lands to be associated with poor cultivation and unfavorable environmental conditions (e.g., unfavourable soil properties), thus soybeans become the primary choice for farmers due to the low cultivation costs and soil benefits. In addition, crop rotation between soybeans and other rain-fed crops has also temporarily added some soybean areas at the local scales. Overall, total soybean gain did not compensate for total soybean loss, because there was a net shrinkage in number and continuity of soybean croplands at all study scales from 2005 to 2010.

Many studies have designated a change in continuity as an important early warning signal of a critical transition in landscapes^26^,^27^,^28^,^29^. Therefore, the decline in continuity of soybean croplands here suggests that former extensively continuous soybean planting areas have been reduced to relatively discrete and isolated patches, which indicate a decline of Heilongjiang non-GM soybeans or even the potential collapse of this crop system.

Because imported GM soybeans are less expensive, many farmers in China have already abandoned the cultivation of non-GM soybeans on their plots and, therefore, displaced domestic soybean production to the soybean-exporting countries of the world^22^,^30^. Many Chinese farmers have switched to other crops, such as corn and rice, or have reverted their plots to forests that could contribute to environmental conservation, such as increased carbon storage and biodiversity restoration^17^,^22^. However, as China’s demand for edible oil and meat continues to soar, a large and steady supply of soybeans (for oil crushing) and soybean meal (for fodder) is strongly needed. In response to this demand, soybeans have become an integral part of agricultural economies in many soybean-exporting countries. For example, both the United States in the northern hemisphere and Brazil in the southern hemisphere provide continuous supplies of soybeans to feed distant consumers like those in China. And this is especially true after the implementation of GM techniques, which simplifies weed control and allows more work flexibility^31^,^32^. Without effective interventions, the non-GM soybean production in Heilongjiang may continue to decline. Because China is the largest producer of non-GM soybeans in the world^22^, and Heilongjiang is China’s largest soybean producer, this decline could be a warning sign to the Chinese and worldwide non-GM soybean industries, impacting all related sectors.

Results from this study confirm the important scientific value and policy implications of the information of the spatiotemporal patterns of non-GM soybean production, as highlighted in the Introduction. These results are important for a variety of policy makers, farmers, and other stakeholders across the world. Information about the spatiotemporal patterns of non-GM soybean production derived from this work could help government agencies (e.g., environmental protection agencies and agricultural extension agencies) to target and manage areas converted from soybeans to corn. It can also encourage the development of new corn varieties with a short growing season, which helps increase the ability to cope with extreme weather events after soybean lands are converted to corn fields. At the same time, our work provides information for cultivation plans in soybean-exporting countries like Brazil. For example, the significant decline in Jiamusi prefecture (a soybean-planting region with high productivity, Supplementary Table 1 and Fig. 3b) signals more soybean demand from China. Therefore, farmers in Brazil and other soybean-exporting countries should make appropriate changes to their cultivation plans in response to this decline in Chinese production.

To make this work more meaningful, we are incorporating the results from this work into a larger interdisciplinary project on telecoupling (socioeconomic and environmental interactions over distances)^17^,^33^. This larger project applies the integrated telecoupling framework to understand and integrate socioeconomic and environmental patterns and processes across various places around the world (e.g., soybean-exporting countries, soybean-importing countries, and other countries affected by the soybean trade)^34^. The project addresses different aspects of telecoupling (e.g., soybean dynamics across China, fertilizer application in China and land change in Brazil in response to the dynamics of soybean production in China). Through systems integration (holistic approaches to integrate various components at different organizational levels and across different scales of coupled human and natural systems)^35^,^36^,^37^,^38^, we can systematically quantify and further enhance the scientific and societal values of spatiotemporal analyses of non-GM soybean production.

## Methods

Crop-specific analyses in China rely heavily on statistics data published in yearbooks, but the yearbooks do not display the detailed spatial distributions of crops. Cropland data interpreted from satellite data are needed to understand changes in crop cover over space. A comprehensive land-cover analysis needs to examine the spatial patterns of changed extents^39^,^40^. Because spatial patterns are often scale-dependent^41^, multi-scale analysis of spatial patterns is necessary to characterise changes in land-cover over a wider spatial range to enable a holistic understanding.

The major cropland data layers of 2005 and 2010, including the locations of soybeans, corn, and rice in Heilongjiang, were created by the Chinese Academy of Agricultural Sciences (Supplementary Fig. 2)^14^. The data were derived from the Moderate Resolution Imaging Spectroradiometer (MODIS) imagery provided daily at 250 m spatial resolution and acquired during the growing seasons (April to October) in 2005 and 2010. Compared to the cropland data layers provided at 56 m and/or 30 m resolution published by the U.S. National Agricultural Statistics Service, 250 m resolution is relatively coarse. However, as farmland plots in Heilongjiang are relatively large and homogeneous, 250 m is an acceptable level of resolution for our study. For each image, pre-processing (registration, calibration, and correction) was performed and the normalized difference vegetation index (NDVI) was calculated. High-quality ground-truth global positioning system (GPS) points for locating specific crops were collected across Heilongjiang for 2005 and 2010; these points play a crucial role in the classification scheme. By stacking the GPS points on the NDVI data, a decision-tree supervised classification, based on the information from crop phenology, crop physiology, and fieldwork data, was undertaken to create crop classification maps. The overall accuracy for the entire province was 87.5% for 2005 and 94.2% for 2010, which was determined by checking the Satellite Pour l’Observation de la Terre (SPOT) images and official statistics yearbooks^14^.

To spatially map the changes in soybean planting area in Heilongjiang, a change detection analysis was first used to map soybean gain and loss at the pixel level between 2005 and 2010 (Supplementary Fig. 3)^42^. Next, the soybean area in 2005, soybean area in 2010, and results of change detection, including soybean gain and loss, were used in the following analysis. A moving window analysis was adopted to characterise the spatial patterns of soybean croplands and their changes, which was conducted at multiple scales by varying the window size, that is, the spatial extent^43^. Specifically, soybean area density was calculated in a predefined rectangular window, which refers to the proportion of soybean pixels in the window. Landscape composition is an important indicator in pattern measurement, and it refers to the abundance of soybean plots represented on the landscape in this work. The soybean area density here can be considered as a normalised metric of landscape composition, which enables people to assess the spatial patterns of soybean planting area. The calculations were repeated in three window sizes – 3.1 km^2^ (7 × 7 pixels), 45.6 km^2^ (27 × 27 pixels), and 410.1 km^2^ (81 × 81 pixels) – to ensure a precise measurement of spatial patterns at multiple scales^44^,^45^. Normally, the candidate window size is selected from a size range, whose upper limit is the entire image size of 473,000 km^2^ here and the lower limit is the image resolution of 0.0625 km^2^. The selection of these three sizes was based on careful examinations that spanned three orders of magnitude, that is, one digit for 3.1 km^2^, two digits for 45.6 km^2^, and three digits for 410.1 km^2^. We set 410.1 km^2^ as the largest window, because the next larger window was too coarse to yield meaningful results.

## Additional Information

**How to cite this article**: Sun, J. *et al.* Spatiotemporal patterns of non-genetically modified crops in the era of expansion of genetically modified food. *Sci. Rep.* **5**, 14180; doi: 10.1038/srep14180 (2015).

## Supplementary Material

Supplementary Information

## Figures and Tables

**Figure 1:**
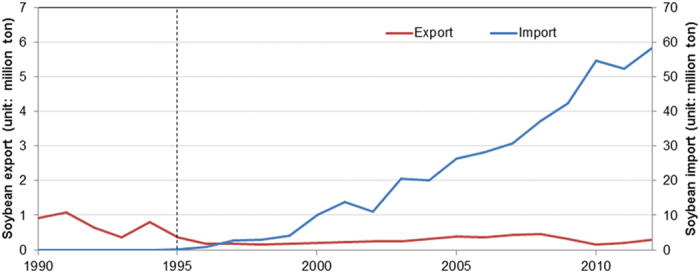
Soybean import/export quantity in China from 1990 to 2012. The vertical dashed line marks the time when soybean imports exceeded exports. Data source: the Statistics Division of the Food and Agriculture Organization of the United Nations at http://faostat.fao.org/.
