# Supplementary material for: Spatiotemporal patterns of non-genetically modified crops in the era of expansion of genetically modified food
Source: Sci Rep. 2015 Sep 18;5:14180. doi: 10.1038/srep14180 (PMC4585609; doi:10.1038/srep14180)
Supplement: Supplementary Information [file srep14180-s1.pdf]

Spatiotemporal patterns of non-genetically modified crops in the era of expansion of genetically modified food

Jing Sun, Wenbin Wu, Huajun Tang, Jianguo Liu

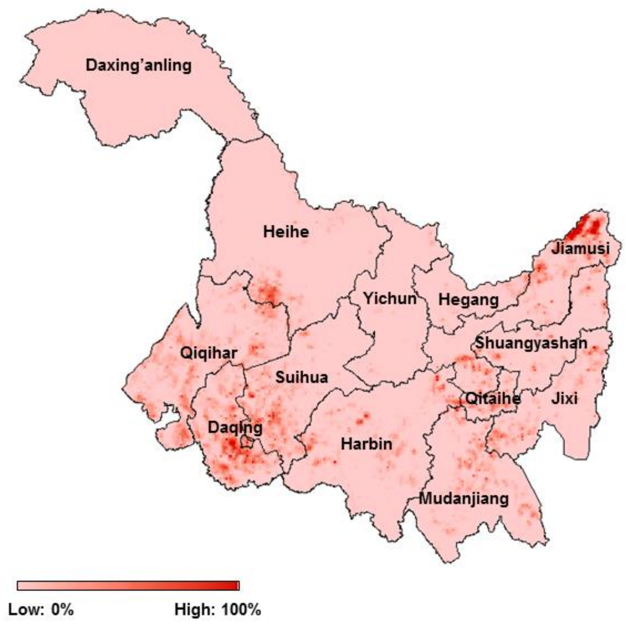

Supplementary Fig. 1 Hotspots of conversion from soybeans to corn and/or rice from 2005 to 2010 in Heilongjiang Province, China. The map was generated by the software ArcGIS 10[1].

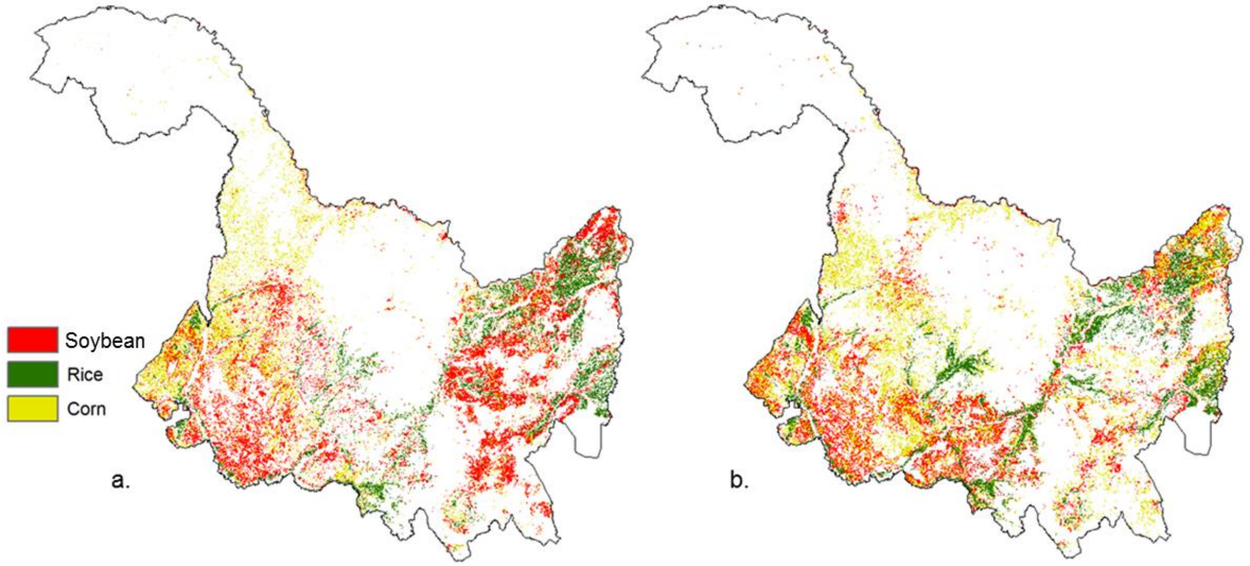

Supplementary Fig. 2 Major crops cover data layers in Heilongjiang Province, China. (**a**) 2005. (**b**) 2010. Other land cover types, including other crops, are not plotted here. The map was generated by the software ArcGIS 10[1].

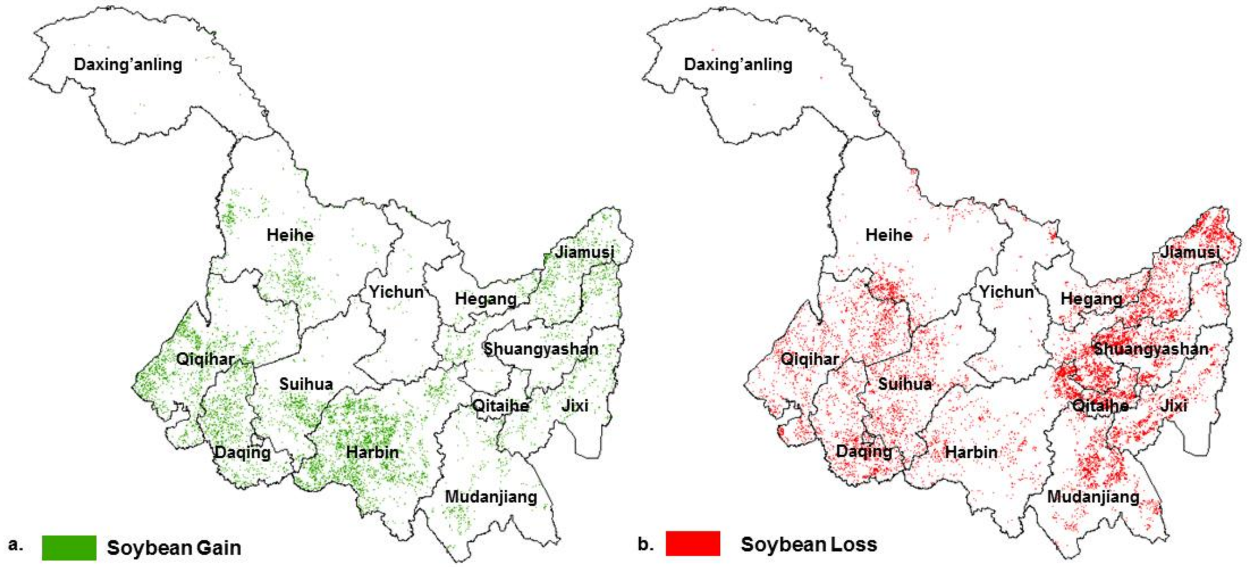

Supplementary Fig. 3 Results of change detection at the pixel level in Heilongjiang Province, China. (**a**) soybean gain. (**b**) soybean loss. Unchanged soybean areas and other land cover types are not plotted here. The map was generated by the software ArcGIS 10[1].

Supplementary Table 1 Average soybean productivity (2000 – 2010) in different prefecture regions of Heilongjiang Province[2]

Rank	Prefecture region	Yield per hectare (ton/hectare)
1	Mudanjiang	2.2
2	Jiamusi	2.1
	Harbin	2.1
	Jixi	2.1
3	Suihua	1.9
	Qitaihe	1.9
4	Heihe	1.8
	Qiqihar	1.8
	Shuangyashan	1.8
5	Yichun	1.7
	Daxing'anling	1.7
6	Daqing	1.6
7	Hegang	1.5

Supplementary Table 2 Greenness date (starting day of growing season counted from January 1st annually) at the prefecture-level regions of Heilongjiang Province, from 2001 to 2012. Data were calculated from MODIS Land Cover Dynamics (MCD12Q2) product, Version 5, provided at 500m.

	Greenness date												
	Daqing	Daxing'anling	Harbin	Hegang	Heihe	Jiamusi	Jixi	Mudanjiang	Qiqihar	Qitaihe	Shuangyashan	Suihua	Yichun
2001	138	124	130	134	127	136	130	130	142	128	129	142	122
2002	136	117	124	132	124	135	126	126	141	125	128	143	118
2003	154	123	129	134	128	142	129	129	155	125	128	145	119
2004	141	129	134	139	131	144	136	136	149	133	136	145	124
2005	143	128	134	139	135	145	137	137	150	136	139	147	131
2006	143	128	132	149	132	147	138	138	149	132	134	143	124
2007	133	120	130	129	125	137	130	130	143	127	129	139	121
2008	138	122	125	134	127	142	129	129	149	128	128	142	118
2009	143	119	128	136	127	146	133	133	154	130	134	145	118
2010	136	120	135	136	127	144	135	135	146	131	137	143	123

Supplementary References

1 ESRI (Environmental Systems Research Institute). *ArcGIS Desktop: Release 10*. (Environmental Systems Research Institute, 2011).

2 HPBS (Heilongjiang Provincial Bureau of Statistics). *Heilongjiang Statistical Yearbook 2000 - 2013*. (China Statistics Press, 2000 - 2013).
